# Influence of Female Genital Mutilation/Cutting on Health Morbidity, Health Service Utilization and Satisfaction with Care among Somali Women and Teenage Girls in the United States

**DOI:** 10.1007/s40615-022-01266-x

**Published:** 2022-03-08

**Authors:** Crista E. Johnson-Agbakwu, Kathleen A. Fox, Aduragbemi Banke-Thomas, Georgia J. Michlig

**Affiliations:** 1 Southwest Interdisciplinary Research Center, Arizona State University, Phoenix, AZ USA; 2 School of Medicine, Creighton University, Phoenix, AZ USA; 3 District Medical Group, Phoenix, AZ USA; 4 School of Criminology and Criminal Justice, Arizona State University, Phoenix, AZ USA; 5 School of Human Sciences, University of Greenwich, London, UK; 6 LSE Health, London School of Economics and Political Science, London, UK; 7 Johns Hopkins Bloomberg School of Public Health, Baltimore, MD USA

**Keywords:** Female circumcision, Health morbidity, Health-seeking behavior, Health equity, Respectful maternity care

## Abstract

**Introduction:**

There is scant evidence on the health morbidities experienced by Somali women and girls affected by female genital mutilation/cutting (FGM/C) and their resultant health-seeking behavior in the USA as compared to those who have not undergone the procedure. To fill this gap, we conducted a comprehensive examination of health morbidity among women and teenage girls with and without FGM/C in a Somali migrant community.

**Methods:**

Using a comprehensive community-based participatory research approach, a cross-sectional survey was administered to 879 Somali women and teenage girls in Phoenix and Tucson, Arizona. We employed Chi-square and analysis of variance to disentangle health and healthcare use among those with and without FGM/C.

**Results:**

The majority of respondents had undergone FGM/C (79%). Respondents with FGM/C experienced significantly more health concerns compared to uncut women and girls, with those possessing Type III FGM/C experiencing significantly more obstetric, gynecologic, sexual, and mental health morbidity than those with Type I or Type II. Rates of service use, while varied, were low overall, particularly for mental health services, even with health insurance. The majority of respondents who sought care indicated that their concerns were resolved, and they were satisfied with the healthcare received.

**Conclusions:**

Community-engaged strategies that build upon satisfaction with care of women who seek care to enhance trust, nurture community embeddedness and facilitate peer navigation, while equipping health and social service providers with the competency and tools to provide respectful, trauma-informed care, will be critical to advance health equity for FGM/C-affected communities.

## Introduction

Female genital mutilation/cutting (FGM/C) is an ancient cultural tradition still currently practiced to varying extents across thirty countries throughout sub-Saharan Africa, South-East Asia, and the Middle East, as well as in some countries across Central and South America. While precise prevalence rates of FGM/C are unknown, it is estimated that over 200 million women and girls have been affected with an estimated 3.6 million girls at risk annually. In the USA, it is estimated that approximately 545,000 women and girls have either undergone a form of FGM/C or were born to women from FGM/C-practicing countries [1]. FGM/C has captured global attention in recent years due to the increasing influx of migrants and refugees to high-income countries where healthcare providers are continually faced with the challenge of providing high quality, culturally sensitive care amidst limited training opportunities on the care of affected women and girls [2, 3].

The World Health Organization (WHO) defines FGM/C as “all procedures that involve partial or total removal of the external female genitalia, or other injury to the female genital organs for non-medical reasons” and classifies FGM/C into categories encompassing varying sub-types that increase in severity, including Type I (clitoridectomy), Type II (Excision) and Type III (infibulation). The WHO also recognizes a fourth type of FGM/C, the least severe form, which encompasses all other procedures to the vulva including: pricking, piercing, incising, scraping and cauterization. Eighty-five percent of all types of FGM/C encompass Types I and II while only 15% comprise Type III [4]. However, war and forced displacement, particularly from countries with high prevalence of FGM/C, has resulted in a disproportionately large number of migrants and refugees with the most severe form of FGM/C (Type III) now residing in high-income countries. For instance, Somalia has among the highest FGM/C prevalence rates in the world with 98% of Somali women having undergone the procedure, with Type III being the most prevalent. From October 1, 2001, to August 31, 2019, 104,000 Somalis have been resettled, representing the single largest African migrant group to be resettled in the USA. [5].

FGM/C-affected women across both high- and low-income countries experience greater health risks including emergency cesarean delivery, postpartum hemorrhage, severe perineal trauma, episiotomy, instrumental vaginal birth, extended maternal hospital stay, low Apgar scores, neonatal resuscitation, stillbirth, and early neonatal death [6–8]. Likewise for gynecologic and sexual morbidity, systematic reviews of genitourinary and sexual sequelae have indicated increased risk of FGM/C-associated morbidity, namely urinary tract infections, bacterial vaginosis, dyspareunia, and attenuation of women’s sexual functioning [9, 10]. A systematic review also reported an association between FGM/C and adverse mental health outcomes [11]. Despite these established linkages between FGM/C and health comorbidity, much remains unknown about FGM/C-related morbidity and the resultant health services use and experiences with care among Somali women. This is the first known large-scale US-based investigation into the following research questions: (1) Do Somali women and teenage girls with FGM/C experience significantly more health concerns than their uncut counterparts? (2) Among those who were cut, when healthcare was used, how often was the concern resolved and were respondents satisfied with the healthcare received? (3) Are there differences in cut participants by the severity of their cut status (e.g., Type I, II, and III)?

## Methods

### Study Design

This project was conducted using a community-based participatory research (CBPR) design in close partnership with the Somali immigrant and refugee community in Phoenix and Tucson, Arizona. The collaboration included bilingual community mobilizers (CMs), representing both the Somali and Somali Bantu communities as well as local Somali religious leaders, medical practitioners, public service agencies serving the Somali community and university researchers. The research was conducted in accordance with prevailing ethical principles, was reviewed and approved by an Institutional Review Board (study #00005252) and conducted with approval by local Somali religious leaders. A comprehensive survey was developed in English by the research team in partnership with Somali CMs, and then forward and back-translated into Somali and Maay Maay^1^ by a private translation company. CMs and other key Somali and Somali Bantu community informants ensured linguistic accuracy and cultural appropriateness. Prior to the survey administration, CMs underwent comprehensive training on human subjects’ protections, confidentiality, privacy and the electronic survey instrument. The sample was generated from a combination of purposive snowball and respondent-driven sampling (RDS) strategies [12]. Specifically, we initially implemented respondent-driven sampling strategies which systematically reduces bias that can arise from non-probability sampling. Our data collection began by meeting the parameters for minimal risk of bias as outlined in standard RDS practice (e.g., respondents accurately report their personal network, recruitment from those networks are random, respondents recruit only a single recruitment). However, data collection was impacted by the 2017 presidential inauguration and the resultant rise in deportations, racial/religious animus, hate crimes against migrants and Muslims and fear of separation of mothers from their daughters, who may have undergone FGM/C, during a time when families were being separated at the USA–Mexico border. The political climate reduced the willingness of Somali migrants to participate in our federally funded project; therefore, we transitioned to purposive snowball sampling which eliminated the restrictions imposed by RDS while maintaining a high level of participation among our target population. CMs verbally administered self-report surveys via an electronic tablet to Somali and Somali Bantu women and teenage girls ages 15 and older from February–December 2017. Parental consent and minor assent were obtained for respondents younger than age 18. Respondents were informed that the survey pertained to FGM/C and healthcare. Although pelvic examinations are often used in medical research to establish the clinical presence of FGM/C, our community-based sample relied on a self-report of FGM/C status, including the severity of cutting, enhanced by a pictorial visual guide of WHO classified FGM/C types that respondents could self-identify, which is consistent with prior research that demonstrates FGM/C is a salient life event clearly recalled by respondents [13, 14].

### Measures

Health morbidity: Respondents were asked if they had experienced a series of 30 general as well as culturally specific health sequela across four dimensions (sexual dysfunction, obstetric, depression/trauma and gynecologic health). Although sexual dysfunction is a form of gynecologic health, we isolated sexual dysfunction as its own category, consistent with prior research [15], given the high prevalence of sexual dysfunction experienced by cut women. Response options included: yes (= 1), no (= 0), unsure (= 3) and not applicable (= 4). Responses were dichotomized into yes and no/unsure/not applicable.

Utilization of health services: For each reported health concern, respondents with any form of FGM/C were asked a series of questions pertaining to whether or not (yes = 1; no = 0) healthcare was used for the problem, the problem was resolved, and satisfaction with healthcare received.

### Statistical Analyses

A series of descriptive statistics are presented pertaining to respondents’ health concerns, health-service utilization and experiences with care. Chi-square analyses were estimated to determine significant differences among cut and uncut women’s health morbidity. Subsequent descriptive analyses focused specifically on respondents with FGM/C. One-way analysis of variance using Bonferroni post-tests featured differences across FGM/C severity/types.

## Results

### Participant Demographics

The sample consisted of 879 Somali women and teenage girls residing in Arizona. Most of our sample were cut (*n* = 687; 78%), and the remainder were uncut (*n* = 161; 18%), whereas only 18 (2%) respondents were unsure of their cut status and recoded as missing data, along with the 13 (1.5%) respondents who did not answer the question. Of the 687 respondents who reported being cut, 605 (88%) also reported the type of FGM/C, 75 were unsure of the type and 38 skipped the question (e.g., missing data). Of the three measured forms of FGM/C that increase in severity, respondents fairly equally self-reported that they had Type I (*n* = 223; 32%), Type II (*n* = 139; 20%) and Type III (*n* = 243; 35%).

In terms of demographic characteristics, respondents ranged in ages from 15 to 90 (Table 1). Uncut women and girls were significantly younger ($\overline{x}$=23) than women with Type II and Type III. Those with Type I were significantly younger ($\overline{x}$=26) than those with Type II ($\overline{x}$=34), who were significantly younger than respondents with Type III ($\overline{x}$=39). Due to uncut respondents’ younger age, most uncut respondents were also single/never married (70%), which was significantly more than those with Type I (53%), Type II (31%) and Type III (19%). In terms of education, uncut respondents were significantly more educated to at least a high school diploma compared to those with Type II and Type III. Women and teenage girls with Type I were also significantly more highly educated than those with Type II and Type III. Those who were uncut had spent significantly more time in the USA ($\overline{x}$= 10.99 years) compared to those with Type I ($\overline{x}$= 8.08 years), Type II ($\overline{x}$= 8.38 years) and Type III ($\overline{x}$= 7.44 years). Many of the respondents were insured, although there were no significant differences across groups (86% of uncut, 87% of Type I, 92% of Type II and 93% of Type III). Notably, the majority of our sample were insured by public insurance, and this finding held consistent across respondents who were uncut (69%) and among those with FGM/C, including those with Type I (79%), Type II (87%) and Type III (88%).

**Table 1:** Sample characteristics among the full sample (*N* = 879) and among women with FGM/C (*n* = 687) and uncut women (*n* = 161)

	Uncut women	Women with FGM/C				
	*n* = 161	Any FGM/C *n* = 687	Type I *n* = 223	Type II *n* = 139	Type III *n* = 243	Significant contrasts
		*n* (%) / Mean (S.D.)				
Age						U < T2, T3; T1 < T2; T1 < T3; T2 < T3
Age in years (range 15–990)	23 (8.86)	33 (13.96)	26 (10.04)	34 (13.47)	39 (14.18)	
Marital status						U > T1, T2, T3; T1 > T2; T1 > T3
Single/never married	112 (70%)	241 (35%)	119 (53%)	43 (31%)	45 (19%)	
Highest level of education attained						U > T2, T3; T1 > T2, T3
Never attended school	27 (17%)	181 (27%)	30 (14%)	44 (32%)	83 (35%)	
Primary school	14 (9%)	122 (18%)	24 (11%)	26 (19%)	60 (25%)	
Some high school	47 (30%)	146 (22%)	63 (29%)	29 (21%)	33 (14%)	
High school diploma	26 (17%)	131 (19%)	52 (24%)	29 (21%)	38 (16%)	
Some college	29 (19%)	60 (9%)	25 (12%)	8 (6%)	18 (8%)	
College/advanced degree	13 (8%)	36 (5%)	23 (11%)	1 (1%)	8 (3%)	
Years in the United States						
Years (range 0–947)	10.99 (6.42)	8.17 (6.84)	8.08 (6.54)	8.38 (5.92)	7.44 (7.71)	U > T1, T2, T3
Insurance status						
Insurance	139 (86%)	614 (89%)	194 (87%)	128 (92%)	226 (93%)	None
No insurance	18 (11%)		27 (12%)	6 (4%)	13 (5%)	
Medicaid/public	111 (69%)		176 (79%)	121 (87%)	215 (88%)	
Private insurance	28 (17%)		18 (8%)	7 (5%)	11 (5%)	

Note: FGM/C = female genital mutilation/cutting. S.D.= standard deviation. One-way analysis of variance using Bonferroni post-tests indicate significant differences (*p* < .05) among uncut women (U) and women with Type I (T1), Type II (T2), and Type III (T3) FGM/C

### Health Morbidity

Women and teenage girls with FGM/C experienced significantly more health morbidity than those who were uncut (Table 2). In terms of sexual dysfunction, cut women were significantly more likely than uncut women to experience delayed intercourse, inability to have intercourse, pain with intercourse, lack of pleasure, lack of sexual desire and bleeding with intercourse. Turning to obstetric issues, cut women were significantly more likely than uncut women to experience difficulty getting pregnant, still birth, emergency Cesarean section (C-section), excessive vaginal tears, postpartum hemorrhage and prolonged hospitalization. This suggests that cut and uncut women may have different obstetric histories. Examining this in a supplemental analysis, we found that among the 161 uncut women, 68 (42%) had never been pregnant, 25 (16%) had multiple pregnancies and another 68 (42%) did not answer the question. Among the 687 cut women, 106 (15%) had never been pregnant, 421 (62%) had multiple pregnancies and 160 (23%) did not answer the question. While exploring these relationships are outside of the scope of this study, it is noteworthy that the available data suggest that uncut women who are younger are more likely to have never been pregnant compared to cut women who are older in our sample. Even so, a substantial number of women, both cut and uncut, did not answer the question about the number of pregnancies and so we are unable to further explore this relationship.

**Table 2:** Association between health morbidity and healthcare experiences among FGM/C (*n* = 687) vs. uncut (*n* = 161) women

	Health morbidity		Healthcare experiences among women with FGM/C		
	FGM/C	Uncut	Healthcare use	Problem resolved	Satisfaction with care
Sexual dysfunction					
Delayed intercourse	40 (6%) *	1 (1%)	15 (38%)	13 (87%)	13 (87%)
Inability to have intercourse	35 (5%) *	1 (1%)	12 (34%)	7 (58%)	10 (83%)
Pain with intercourse	97 (15%) *	2 (2%)	28 (29%)	24 (86%)	24 (86%)
Lack of pleasure during sex	73 (11%) *	4 (3%)	27 (37%)	23 (85%)	23 (85%)
Lack of sexual desire	60 (9%) *	1 (1%)	26 (43%)	20 (77%)	21 (81%)
Bleeding with intercourse	55 (9%) *	1 (1%)	21 (38%)	19 (90%)	19 (90%)
Obstetric issues					
Difficulty getting pregnant	22 (4%) *	0	13 (59%)	7 (54%)	7 (54%)
Infertility	12 (2%)	1 (1%)	7 (58%)	3 (43%)	4 (57%)
Post-term pregnancy	20 (3%)	1 (1%)	10 (50%)	10 (100%)	9 (90%)
Stillbirth	29 (5%) *	0	17 (59%)	14 (82%)	15 (88%)
Fetal distress	15 (2%)	0	11 (79%)	9 (82%)	9 (82%)
Emergency C-section	29 (5%) *	0	24 (83%)	23 (96%)	22 (92%)
Neonatal resuscitation	9 (2%)	0	8 (89%)	8 (100%)	6 (75%)
Extensive vaginal tears	34 (5%) *	0	22 (65%)	20 (91%)	18 (82%)
Postpartum hemorrhage	19 (3%) †	0	15 (79%)	15 (100%)	14 (93%)
Prolonged hospitalization	24 (4%) *	0	20 (83%)	19 (95%)	20 (100%)
Mental health sequelae					
Feeling sad for many weeks	41 (6%) *	0	16 (39%)	14 (88%)	14 (88%)
Having flashbacks/nightmares about traumatic event	29 (5%) †	1 (1%)	6 (21%)	5 (83%)	4 (67%)
Gynecological sequelae					
Pain with menstruation	129 (20%) *	7 (6%)	58 (45%)	42 (72%)	46 (79%)
Difficulty passing menstrual blood	73 (11%) *	0	31 (42%)	19 (61%)	22 (71%)
Prolonged menstruation	24 (4%) †	1 (1%)	14 (58%)	10 (72%)	9 (64%)
Other recurrent bleeding	10 (2%)	0	6 (60%)	4 (67%)	4 (67%)
Difficulty passing urine	29 (5%) *	1 (1%)	11 (38%)	7 (64%)	9 (82%)
Pain with urination	36 (6%) *	0	13 (36%)	8 (62%)	11 (85%)
Recurrent urinary tract	21 (3%) *	0	17 (81%)	15 (88%)	15 (88%)
Vaginal itching	31 (5%) *	1 (1%)	19 (61%)	17 (89%)	15 (79%)
Recurrent genital infections	18 (3%)	1 (1%)	15 (83%)	12 (80%)	11 (73%)
Cysts in genital area	7 (1%)	1 (1%)	6 (86%)	3 (50%)	4 (67%)
Scarring in genital area	29 (5%) *	1 (1%)	9 (31%)	6 (67%)	6 (67%)
Fistula	1 (1%)	0	1 (100%)	0	0

FGM/C = female genital mutilation/cutting *p < .05; †*p* < .10

Both measures of mental health sequela were experienced significantly more among cut compared to uncut women (e.g., feeling sad for many weeks and having flashbacks/nightmares about a traumatic event). Cut women also suffered from significantly more gynecologic sequela compared to uncut women, including pain with menstruation, difficulty passing menstrual blood, prolonged menstruation, difficulty passing urine, pain with urination, recurring urinary tract infections, vaginal itching and genital scarring. To better understand the healthcare experiences of cut women and teenage girls, the remaining analyses focus exclusively on cut participants.

### Health Service Utilization

Beginning with sexual dysfunction, fewer than half of the cut women used services, yet many of the women who used available services had their concern resolved and were satisfied with the care received. Although the data on health service utilization are too extensive to feature entirely, an illustrative example of this pattern can be observed among cut women experiencing pain with intercourse. Specifically, only 29% of cut women experiencing pain with intercourse used health services; yet 86% of those who sought care had their problem resolved and were satisfied with the care received (Table 2).

The majority of cut women and teenage girls experiencing obstetric issues used health services, had their concern resolved and were satisfied with the care they received. This pattern holds across all obstetric concerns; although, the pattern is weakest among cut participants with infertility (58% used services, 43% of those who used services had the problem resolved, and 57% of those who used services were satisfied with the care they received). As an example of this pattern with high prevalence rates, cut women who underwent an emergency C-section (83% used services, 96% of those who used services had the problem resolved and 92% were satisfied with the care received) (Table 2).

Cut women experiencing mental health concerns were unlikely to use health services (39% of those who felt sad for weeks and 21% of women experiencing flashbacks/nightmares). However, cut women who used services for mental health concerns reported having their problem resolved (88% and 83%, respectively) and being satisfied with the care they received (88% and 67%, respectively) (Table 2).

Service utilization among cut participants varied considerably by type of gynecologic sequela. On the lower end of the spectrum, cut women and teenage girls were less likely to use services for pain with menstruation (45%), difficulty passing menstrual blood (42%), difficulty passing urine (38%), pain with urination (36%) and genital scarring (31%). Alternatively, the majority of cut participants used health services for prolonged menstruation (58%), other recurrent bleeding (60%), recurrent urinary tract infection (81%), vaginal itching (61%), recurrent genital infections (83%) and genital cysts (86%). Regardless of gynecologic health morbidity, the majority of cut women and teenage girls who used services had their concern resolved and were satisfied with care.

### Differences across FGM/C type Severity

Women and teenage girls with the most severe form of FGM/C (Type III) experienced higher rates of all 30 measured health sequela compared to participants with less severe cutting forms. Women and teenage girls with Type III cutting were significantly more likely than those with Type I and Type II to experience sexual dysfunction, obstetric, mental health and gynecologic concerns (Table 3).

**Table 3:** Health morbidity among cut women by FGM/C severity/type

Health morbidity	Any FGM/C *n* = 687	Type I *n* = 223	Type II *n* = 139	Type III *n* = 243	Significant contrasts
Sexual dysfunction					T1 < T3; T2 < T3
Delayed intercourse	40 (6%)	12 (5%)	2 (1%)	25 (10%)	
Inability to have intercourse	35 (5%)	6 (3%)	6 (4%)	20 (8%)	
Pain with intercourse	97 (15%)	18 (8%)	11 (8%)	61 (25%)	
Lack of pleasure during sex	73 (11%)	15 (7%)	9 (6%)	45 (19%)	
Lack of sexual desire	60 (9%)	14 (6%)	12 (9%)	33 (14%)	
Bleeding with intercourse	55 (9%)	7 (3%)	9 (6%)	36 (15%)	
Obstetric issues					T1 < T3; T2 < T3
Difficulty getting pregnant	22 (4%)	3 (1%)	3 (2%)	11 (5%)	
Infertility	12 (2%)	2 (1%)	2 (1%)	8 (3%)	
Post-term pregnancy	20 (3%)	5 (2%)	3 (2%)	11 (5%)	
Stillbirth	29 (5%)	1 (< 1%)	4 (3%)	21 (9%)	
Fetal distress	15 (2%)	3 (1%)	1 (1%)	10 (4%)	
Emergency C-section	29 (5%)	7 (3%)	3 (2%)	14 (6%)	
Neonatal resuscitation	9 (2%)	1 (< 1%)	2 (1%)	5 (2%)	
Extensive vaginal tears	34 (5%)	6 (3%)	9 (6%)	17 (7%)	
Postpartum hemorrhage	19 (3%)	2 (1%)	5 (4%)	12 (5%)	
Prolonged hospitalization	24 (4%)	3 (1%)	2 (1%)	18 (7%)	
Mental health sequelae					T1 < T3
Feeling sad for many weeks	41 (6%)	5 (2%)	11 (8%)	23 (9%)	
Having flashbacks/nightmares about traumatic event	29 (5%)	6 (3%)	7 (5%)	12 (5%)	
Gynecological sequelae					T1 < T3; T2 < T3
Pain with menstruation	129 (20%)	34 (15%)	17 (12%)	70 (29%)	
Difficulty passing menstrual blood	73 (11%)	12 (5%)	9 (6%)	49 (20%)	
Prolonged menstruation	24 (4%)	8 (4%)	1 (1%)	15 (6%)	
Other recurrent bleeding	10 (2%)	1 (< 1%)	0	8 (3%)	
Difficulty passing urine	29 (5%)	6 (3%)	2 (1%)	19 (8%)	
Pain with urination	36 (6%)	5 (2%)	3 (2%)	24 (10%)	
Recurrent urinary tract	21 (3%)	3 (1%)	4 (3%)	11 (5%)	
Vaginal itching	31 (5%)	7 (3%)	4 (3%)	16 (7%)	
Recurrent genital infections	18 (3%)	4 (2%)	3 (2%)	9 (4%)	
Cysts in genital area	7 (1%)	0	2 (1%)	5 (2%)	
Scarring in genital area	29 (5%)	7 (3%)	5 (4%)	15 (6%)	
Fistula	1 (1%)	0	0	1 (< 1%)	

FGM/C = female genital mutilation/cutting. One-way analysis of variance using Bonferroni post-tests indicate significant differences among women with Type I (T1), Type II (T2) and Type III (T3) FGM/C

## Discussion

Our findings demonstrate that women with increasing severity of FGM/C experienced more obstetric, gynecologic and sexual morbidity, which aligns with existing literature [6]. However, despite the high morbidity, FGM/C-affected respondents largely underutilized healthcare services. Of the three morbidity categories, service utilization was lowest for sexual dysfunction. Prior research shows that women often experience untoward encounters in their interactions with healthcare providers which only hinders open, respectful, trust-based and non-judgmental dialogue [16]. It is possible that low service utilization in this population is related to poor prior healthcare experiences. Moreover, confusion, misinformation, ambiguity and unfamiliarity with navigating the legal and ethical conundrums that often arise concerning one’s migration status, the persistent geo-political milieu of racial and migrant animus [17], US laws criminalizing the practice of FGM/C, and imprecise estimates of girls at risk [1], alongside the looming threat of engagement with child protective agencies, often drive families to delay or avoid interactions with the healthcare system [18].

For mental health in particular, FGM/C-affected women and teenage girls also experienced more mental health sequela compared to uncut women and teenage girls. However, the relationship between FGM/C and mental wellbeing is complex and while no causal inference between FGM/C and mental health can be specifically inferred [19], reported service use was particularly low for mental health care in our study. In addition to the factors which create barriers to care utilization discussed for physical health, mental healthcare utilization may be additionally influenced by the strong stigma surrounding mental health which remains pervasive in the community [20]. In addition, cultural perceptions of mental illness have been linked to reduced care utilization for mental health issues [21].

Previous studies have highlighted the effect of having health insurance on care seeking and service utilization [15, 22, 23]. Despite a majority of respondents in this study possessing a form of health insurance, in many cases, Medicaid, it does not appear that this alone leads to adequate health services use as unmet care needs persist. However, the care package offered also matters. For example, Assisted Reproductive Technology (ART) is a service that is not covered under Medicaid [24], and given that most of our sample were insured by Medicaid, many of our sample may find infertility treatments prohibitive due to high out-of-pocket costs. This might partly explain the underutilization observed for infertility issues.

Among the women and teenage girls who sought care, the majority had their concerns resolved satisfactorily. However, among FGM/C-affected respondents with infertility, their concerns largely persisted with unsatisfactory resolution. This calls for more research to understand what needs to be done to improve satisfaction with care among those with infertility. An interesting, yet counter-intuitive observation was the high rate of satisfaction with care (92%) among participants who underwent emergency cesarean delivery, despite the profound fear and avoidance of cesarean delivery that is replete in the literature [25–28]. This might suggest that when left to the women, they would rather not have a C-section, yet, regardless of mode of delivery, if they had a successful outcome, they may be “satisfied” with the care rendered. In earlier findings published from the same research project, our team found that perceived discrimination was associated with reduced satisfaction in care [23].

To our knowledge, this is the largest US-based study to examine health morbidity and health service utilization among Somali women and teenage girls, containing among the most comprehensive and extensive measures of health sequela. There are other strengths of this study, namely the inclusion of adolescents as young as age 15, as well as the inclusion of a large sample of women and teenage girls without FGM/C which allows for congruent comparisons with FGM/C-affected respondents along homogenous ethnocultural identity and migratory journeys. Furthermore, respondents self-report of FGM/C status was aided by the incorporation of FGM/C visual imagery using an electronic tablet, the accuracy of which was further strengthened given the high prevalence of the most extensive form of FGM/C (Type III) in this sample, which is consistent with the high rate of FGM/C among women and girls in Somalia (98%) [13, 29]. This is notable given the high unreliability of self-reported FGM/C status in community and population-based studies where clinical genital examination is not feasible or permissible [30]. In addition, the principles of CBPR undergirded the very foundation of this study’s successful implementation in a community with a longstanding history of partnership, nurtured trust, and sustained community embeddedness during a perilous time in US history amidst racial, migrant and religious hostility, and on a highly sensitized and taboo subject matter wherein communities would rather remain hidden [17]. However, this study is not without its limitations, principally that our findings may not be generalizable to other Somali and/or FGM/C-affected communities across the USA, especially if the quality of care available to them is different to that investigated in our study. Consistent with other research that employs cross-sectional and non-probability sampling, our study is unable to infer causal relationships between FGM/C and health morbidity, because of temporality. Furthermore, given the comprehensiveness of the measures of health morbidity assessed, coupled with splitting the sample into uncut vs. the different cut types, the base rates across many cells were below the size needed to perform further tests of significance. Varying demographics and life experiences between sampled cut and uncut women suggests that social differences may bring as much to bear on the morbidity described in this study as the FGM/C itself. That uncut women were more likely to be younger, more highly educated and with longer lives spent in the USA may be an important consideration in contextualizing future research.

There are important implications from this study for future health policy and practice. Strategies to increase health-seeking and service utilization, and to address mental health stigma must engage communities in robust, yet meaningful partnerships where trust can be nurtured and sustained [16]. One such strategy may involve the formation of a Community Advisory Board comprised of community stakeholders representing women, men, youth, elders and religious leaders, which informs the healthcare, programmatic and research priorities targeting FGM/C-affected communities [18]. Peer navigators (*Community Health Workers* or *Cultural Health Navigators*) may foster trust, peer mentorship and support, as well as enhance health literacy and facilitate care coordination and referrals to wrap-around services, minimizing loss to follow-up and gaps in care [31, 32]. A key message that these bridging navigators can share with the community is the reassurance that their problems will be resolved, and they will generally be satisfied with care that they can expect from services received, as our study has shown. It is also crucial for healthcare providers to receive iterative training on trauma-informed care approaches, which are culturally informed and linguistically congruent [2, 3, 16]. Training must be extended beyond clinicians to also engage mental health and social service providers [20].

## Conclusion

FGM/C-related morbidity must be disentangled from the maternal morbidity pervasive within the racialized lens through which FGM/C-affected women, who also happen to be Black, Indigenous, People of Color (e.g., BIPOC), often experience care in the USA, and for which FGM/C may serve as a cultural marker for worsened health inequities [16, 33]. Respectful maternity care has garnered considerable attention in recent years, emerging as an anti-racist framework for ensuring equitable attention to the patient experience as a conduit for quality improvement in care and outcomes [34]. As the US accelerates the pace of refugee new arrivals [35], attention must be paid to implementing sustainable policies that promote greater health-seeking behavior, health service utilization, and respectful experiences with care, and advances health and racial equity for the most vulnerable of populations.

## Data Availability

Data and materials can be made available upon request.

## Footnotes

1 Maay Maay is an oral language primarily spoken by Somali Bantus. Although the Somali language is universally spoken by Somalis and Somali Bantus alike, it was of high cultural importance for our project to offer the survey in Maay Maay which clearly communicated our inclusion of the Somali Bantu community. Only two respondents completed the survey in Maay Maay. The remaining respondents completed the survey in English (*n* = 727; 83%) and Somali (*n* = 150; 17%).
